# Temperature-Dependent Compatible and Incompatible Pollen-Style Interactions in *Citrus clementina* Hort. ex Tan. Show Different Transglutaminase Features and Polyamine Pattern

**DOI:** 10.3389/fpls.2020.01018

**Published:** 2020-07-08

**Authors:** Iris Aloisi, Gaetano Distefano, Fabiana Antognoni, Giulia Potente, Luigi Parrotta, Claudia Faleri, Alessandra Gentile, Stefania Bennici, Lavinia Mareri, Giampiero Cai, Stefano Del Duca

**Affiliations:** ^1^ Department of Biological, Geological and Environmental Sciences, University of Bologna, Bologna, Italy; ^2^ Department of Agricultural and Food Production Sciences, University of Catania, Catania, Italy; ^3^ Department for Life Quality Studies, University of Bologna, Rimini, Italy; ^4^ Department of Life Sciences, University of Siena, Siena, Italy

**Keywords:** *Citrus clementine*, plant reproduction, pollen tube growth, self-incompatibility, polyamines, transglutaminase

## Abstract

In clementine, failure of fertilization can result in parthenocarpic fruit development, which has several advantages, such as seedless fruit, longer shelf-life, and greater consumer appeal. Recently, S-RNases have been identified in *Citrus grandis*, thus revealing that the self-incompatibility (SI) reaction relies on the S-RNase gametophytic mechanism. The fundamental role of environmental factors, mostly temperature, in determining the numbers of pollen tubes reaching the ovary is also well established in *Citrus*. In the present work, temperature-dependent pollen–pistil interactions in *C. clementina* were analyzed, focusing on several morphological aspects, as well as on polyamine (PA) content and the activity and distribution of transglutaminase (TGase), both reported to be involved in the SI response in pear and in pummelo. Results clearly indicate that temperature contributed to a different activation of the SI response, which occurs at optimal temperature of 25°C but was by-passed at 15°C. TGase activity was stimulated during the SI response, and it localized differently in the compatible and incompatible interaction: in compatible pollinated styles, TGase localized inside the style canal, while it was detected all around it in incompatible crosses. TGase localization and activity were congruent with the levels of soluble and insoluble conjugated PAs and with morphological evidences, which highlighted cell wall modification occurring as a result of SI.

## Introduction

In order to avoid self-fertilization, plants have adopted different strategies, among which, the asynchronous development of male and female organs, their specific localization within the flower or in the tree crown, and genetics-based strategies (Del Duca et al., 2019), the latter defined as “self-incompatibility” (SI). SI consists in the rejection of pollen from genetically related individuals (“self” pollen) thus preventing inbreeding and finally promoting outcrossing. Based on the pollen grain genome involved in the SI response, it can be gametophytic SI (GSI) or sporophytic SI (SSI) (Fujii et al., 2016). Even though the final result is the rejection of pollen, in GSI, growth of self-pollen tubes is initially allowed but blocked further along the style, while pollen rejection occurs on the stigma in SSI, and pollen tube growth is not allowed at all, and in some cases not even pollen grain rehydration (Mcclure and Franklin-Tong, 2006).

GSI strategies are controlled by a single polymorphic locus called the *S* locus, expressed in several combinations, both in the pistil and in the pollen grain. However, several other genes, proteins, and external factors are involved in the process of pollen rejection/acceptance (Serrano et al., 2015; Qu et al., 2017). To date, two GSI systems have been well characterized, one in Papaveraceae and the other in various families, including Solanaceae, Plantaginaceae, and Rosaceae. In the latter, the stylar *S* locus encodes for ribonuclease glycoproteins (S-RNases), which are taken up by the pollen tube. In compatible pollen, S-RNases are degraded, while they remain active in the incompatible one, causing the degradation of pollen RNA and leading to the block of pollen tube growth and to programmed cell death (PCD) as demonstrated in Papaveraceae (Thomas et al., 2006; Wilkins et al., 2014) and, more recently, in the Malineae (Wang et al., 2010; Li et al., 2018).

The genus *Citrus* contains both self-incompatible and self-compatible species (Masashi et al., 2006). A peculiar aspect of this genus is that the combination of SI and parthenocarpy results in seedlessness, an aspect appreciated by consumers. Therefore, the study of SI in *Citrus* is of great interest for both commercial purposes and for understanding how the SI system has evolved in this genus. In *Citrus*, self-incompatible pollen grains germinate, and pollen tubes grow through the stylar tissue before being blocked (Soost, 1965). The incompatibility reaction has been reported to occur at different stages of pollen tube growth in different *Citrus* species (Distefano et al., 2009). An increasing number of studies have recently focused on the SI mechanism in *Citrus*. Comparative transcriptome approaches have been applied in order to identify proteins putatively involved in the SI response; however, only in *C. grandis* L. Osbeck (pummelo), an S-RNase gene (homolog to those of Rosaceae and Solanaceae) has been identified, whose product was able to inhibit pollen tube growth (Liang et al., 2017). Recently, the S-RNase-based SI system was verified in pummelo, and S-RNases were confirmed to be the pistil S determinants (Liang et al., 2020).

Pollen performance was recently correlated not only to its genotype, but also to temperature during the progamic phase (Distefano et al., 2012). This was highlighted in three *Citrus* species, among which was *C. clementina*, a species that mainly produces seedless fruits, but maintains the capacity to produce seeds when cross-pollinated. Temperature appears to influence the SI reaction by affecting the distance at which pollen tubes are blocked (Distefano et al., 2012). At an optimal temperature of 25°C, the SI response occurred regularly, and pollen tube growth was blocked inside the style, whereas in cold conditions (15°C) the SI response did not occur (Distefano et al., 2018). A recent study indicated how temperature stresses during the flowering stage in *Citrus *affect male gametophyte development, resulting in a drastic reduction in pollen performance (Bennici et al., 2019). The study of SI mechanisms in relation to temperature is also interesting in the light of a climate change scenario. In fact, both temperate and tropical species showed an erratic setting of both seeds and fruits when exposed to temperatures outside the optimal values during the flowering phase (Hedhly, 2011).

Among the factors involved in the SI response, transglutaminase (TGase) was demonstrated to play a relevant role (Del Duca et al., 2010; Gentile et al., 2012). TGase catalyzes post-translational modifications of proteins, through the incorporation of primary amines or *via* protein cross-linking (Aloisi et al., 2016b). These enzymes are widespread in living organisms, share a conserved catalytic site (Makarova et al., 1999; Del Duca and Serafini-Fracassini, 2005; Serafini-Fracassini et al., 2009), and have also biotechnological applications (Scarnato et al., 2016). Its activity was shown to increase during the SI response (Del Duca et al., 2010; Gentile et al., 2012), and this stimulation was mainly due to an increase in Ca^2+^ levels (Della Mea et al., 2007). Increased enzyme activity leads to the cross-link of several substrates, *e.g.* cytoskeleton proteins, and in the post-translational modifications of proteins through their binding to PAs (Di Sandro et al., 2010; Aloisi et al., 2016a), thereby affecting pollen tube growth.

In plants, the most abundant PAs, *i.e.,* putrescine (Put), spermidine (Spd), and spermine (Spm), are small cationic molecules essential for normal cell growth; their content is finely tuned by biosynthesis, degradation, conjugation, and transport (Antognoni et al., 1999; Romero et al., 2018). They also play a role in the fertilization process (Aloisi et al., 2017), acting directly as signaling molecules or as structuring molecules after cross-linking to proteins and cell wall components by the enzyme TGase (Del Duca et al., 2014; Aloisi et al., 2016a). An increase in PA content is fundamental during pollen tube emergence and elongation, as inhibitors of the PA biosynthetic pathway drastically impair its germination (Antognoni and Bagni, 2008). During the progression of pollen tube elongation, the balance between free and bound PAs is modulated so that PAs bound to low- and high-mass molecules (*e.g.*, phenolic compounds and proteins or cell wall components, respectively) increase, while the content of free PAs decreases (Antognoni and Bagni, 2008). Because of their release in the extracellular matrix of the pollen tube (Bagni et al., 1981), they have been hypothesized to modulate RNase activity during tube emergence and growth (Speranza et al., 1984). Being substrates for PA oxidases, whose activities generate H_2_O_2_, PAs in the apoplast are involved in the regulation of cell wall deposition and in wall stiffening (Wu et al., 2010; Aloisi et al., 2015; Cai et al., 2015; Fraudentali et al., 2020).

In light of the importance of TGase and PAs during pollen tube growth and the SI response, TGase activity and distribution, profiles of free, soluble-conjugated and insoluble-bound PAs were investigated in styles of *C. clementina*, a well-known reference for the SI test (Distefano et al., 2018; Bennici et al., 2019). *C. clementina* was self- and cross-pollinated under two different temperature regimes (25°C and 15°C) in order to investigate whether and how temperature can affect pollen rejection.

## Material and Methods

### Materials

All reagents, unless otherwise indicated, were purchased from Sigma Aldrich (Milano, Italy).

### Plant Material

**Figure 1:**
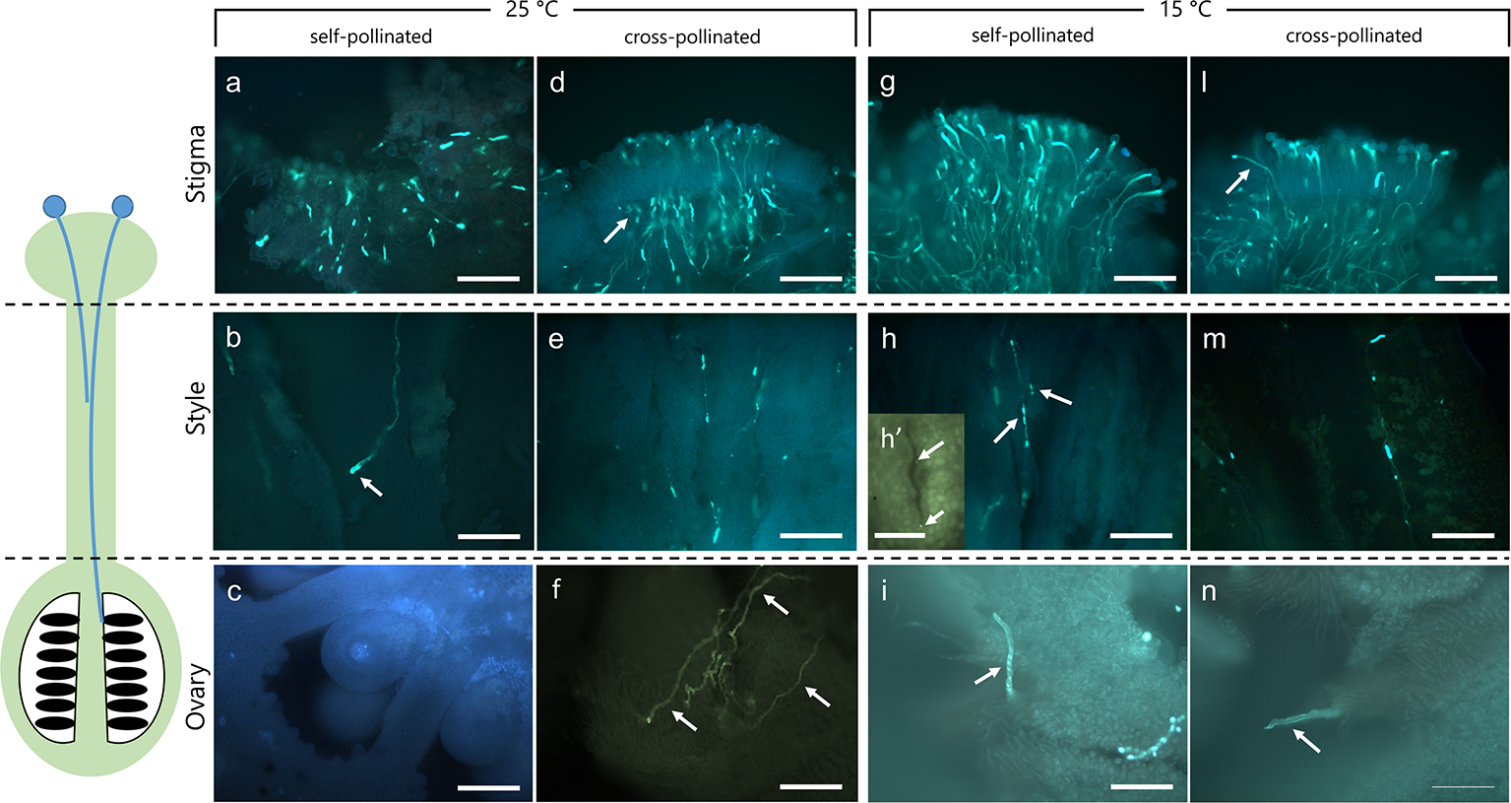
Dissection of pistils in compatible and incompatible crosses at 25°C and 15°C. Aniline blue staining of self-pollinated (incompatible crossing) and cross-pollinated (compatible crossing) pistils at 25°C **(A–F)** and 15°C **(G–N)**. **(A)** In the incompatible crosses at 25°C the stigmatic region showed a few elongating pollen tubes (arrow). **(B)** Pollen tubes were observed in the stylar canals close to the stigma. Pollen tube growth stopped in the upper part of the style (arrow). **(C)** In the ovary, pollen tubes were absent. **(D)** In compatible crosses at 25°C, numerous pollen tubes were observed in the stigma (arrow). **(E)** Pollen tubes grew along the style, reaching the ovary **(F)** (arrows). **(G)** Stigma of self-incompatible cross at 15°C showing numerous pollen tubes. **(H)** Median portion of styles as observed in cross-section. Stylar canals have several pollen tubes (arrows) as also highlighted by the cross-sectioned stylar canal in H’. **(I)** Ovary region where pollen tubes were observed. **(L)** In compatible crosses at 15°C, some pollen tubes (arrow) were evident in compatible stigmas and along the style **(M)**, reaching the ovary (**N**, arrow). Scale bars: 200 μm, except for **(H)**’ (100 µm).

Four-year-old *C. clementina* Hort. ex Tan. plants were grown in pots inside growth chambers and subjected to two different temperatures (15 or 25°C) as previously reported (Distefano et al., 2018; Bennici et al., 2019). Temperature was applied from the appearance of the first flower buds (**Figure 1**, **Supplementary Material 1**) until the end of anthesis. At least three plants were analyzed for each treatment.

### *In Vivo* Pollen Tube Growth

A batch of 50 flowers randomly selected from the three replicate plants grown at 15 or 25°C were self- and cross-pollinated one day before anthesis. Flowers of clementine were emasculated at balloon stage, hand-pollinated with a small paint brush (10 flowers per treatment) and bagged in cotton tissue. Ten days after pollination, 10 pistils were collected. Unpollinated pistils were collected and used as reference material. Pistils were randomly chosen to create pooled samples of 10 pistils. These were freeze-dried until tissue extraction. Some samples were fixed in FAA solution (formalin/glacial acetic acid/70% ethanol, 1:1:18, v/v), and maintained at 4°C until processing for microscopic observation and immunostaining for TGase and staining for callose. Four biological replicates of each sample were analyzed.

### HPLC Analysis of Polyamines

PA extraction and analysis by HPLC were performed as recently described (Mandrone et al., 2019). Briefly, dried styles were extracted in 100 vol. of 4% (w/v) cold perchloric acid (PCA) for 1 h at RT, then centrifuged at 15,000× g for 30 min at 4°C. Supernatant was measured and used for analysis of Free (F) and soluble-conjugated (SC) PAs. After washing the pellets twice with cold PCA, it was resuspended in the original volume and used for analysis of insoluble-bound (IB) PAs. SC and IB fractions were subjected to hydrolysis with 6N HCl for 24 h at RT, in order to free polyamines from their conjugates (Torrigiani et al., 1989) thus dansylated according to Smith and Davies (Smith and Davies, 1985) with minor modifications.

Standard polyamines and heptamethylendiamine, as an internal standard (all by SIGMA-Aldrich), were subjected to the same procedure.

HPLC analysis was carried out on a Jasco system (Jasco—Tokyo, Japan) consisting of PU-4180 pump, FP-821 detector, and an AS-4050 autosampler. The stationary phase was an Agilent (Santa Clara, CA, USA) Zorbax Eclipse Plus C18 reversed-phase column (100 mm × 3 mm I.D., 3.5 μm), and the mobile phase was a mixture of acetonitrile and water. Elution was carried out as previously described (Mandrone et al., 2019). Eluted peaks were detected by the FP fluorimeter at 365 nm excitation and 510 nm emission, and data signals were acquired and processed through the software Chromnav 2.0 (Jasco). Results were expressed as nmol/g DW.

### Protein Extraction, TGase Activity Assay and ELISA-Based Quantification of Isopeptide (N*ε*-(**γ**-Glutamyl)-Lysine) Content

Pistil proteins were extracted as recently reported (Mandrone et al., 2019) with minor modifications. Briefly, unpollinated and pollinated pistils were ground in liquid nitrogen and proteins extracted by stirring for 30 min at 4°C in extraction buffer (40 mg/ml) containing 100 mM Tris–HCl pH 8.5, 10 mM 2-mercaptoethanol, 0.2% Triton X-100, and protease inhibitor cocktail. After centrifugation at 12,000 rpm for 10 min at 4°C, protein concentration in the supernatant was determined by the 2-D Quant Kit (GE Healthcare) according to the manufacturer’s protocol. Extraction was repeated in triplicate and extracted proteins checked by SDS-PAGE.

*In-vitro* TGase activity was measured by the conjugation of biotinylated cadaverine to exogenous substrates *N*, *N*′-dimethylcasein as previously described. Specific activity was determined as a change in A450 of 0.1 per hour per mg of pollen after subtraction of the value of the controls treated with 20 mM EGTA.

ELISA assay was carried out as described previously (Paris et al., 2017). Briefly, a 96-well plate was incubated overnight at 4°C with extracted proteins (50 μl/well, corresponding to 50 μg protein/well). Wells were washed twice with PBS buffer, then incubated 1 h at RT with 200 μl/well of 5% defatted dry milk dissolved in PBS. Wells were washed twice and the mouse monoclonal anti-N^*ε*^-(**γ**-glutamyl)-lysine antibody [81D4] (Covalab, Villeurbanne, France) was added after dilution (1:100) in PBS for 2 h at RT. Then, wells were washed three times with 0.05% Tween 80 in PBS and then incubated with an anti-mouse immunoglobulin conjugated to peroxidase antibody (1:500) for 1 h at RT. After washing, the substrate solution of 0.3 mM 3,3′,5,5-tetramethylbenzidine (TMB) (10 mg/ml in dimethyl sulfoxide) and 0.03% (v/v) hydrogen peroxide in 100 mM sodium acetate pH 6.0 was added. The staining development was stopped after maximum 30 min with 50 μl per well of 5 N H_2_SO_4_. The absorbance value was read at 450 nm using a Tecan Infinite 200 PRO (Tecan, Männedorf, Switzerland) spectrophotometer. As positive control, casein cross-linked *via* TGase was tested in the same conditions. Casein (final concentration 2 mg/ml) cross-linking was carried out by incubating for 2 h in 50 mM Tris–HCl pH 7.4, 150 mM NaCl, 2.5 mM CaCl_2_, and 10 mM DTT in the presence of 5 μg of guinea pig liver (gpl) TGase. The final volume of the reaction was 250 μl. As negative control, casein was incubated in the same conditions with the omission of gpl TGase.

### Immunolocalization of TGase

Compatible and self-incompatible pistils were directly thawed in a buffer solution (100 mM Pipes pH 6.8, 10 mM EGTA, 10 mM MgCl_2_, 0.1% NaN_3_) plus detergent and fixative (0.05% Triton X-100, 1.5% paraformaldehyde, 0.05% glutaraldehyde) for 30 min on ice and then at 4°C for an additional 30 min. For the localization of TGase, fixed pistils were cut along their length and placed in the buffer solution containing 0.75% cellulysin and 0.75% pectinase for 7 min. For immunofluorescence microscopy, samples were washed in the above buffer and incubated with the anti-TGase antibody Ab3 (Neomarker, Fremont, CA, USA) diluted 1:20 in the buffer; incubation was 1 h at 37°C according to previous literature (Del Duca et al., 2009). After washing with buffer, pistils were incubated with the Alexa-Fluor 488-conjugated goat anti-mouse (Thermo Fisher Scientific) secondary antibody diluted 1:50 in the buffer solution, for 45 min at 37°C in the dark. Samples were observed with a Zeiss Axiophot fluorescence microscope (Ex-Max 490 nm/Em-Max 525 nm) equipped with a MRm video camera and a 63× oil-immersion objective.

### Callose Staining and Fluorescence Microscopy Analysis

Unpollinated, cross-compatible, and self-incompatible pollinated pistils were cut longitudinally and transversally 10 days after pollination, then were stained with 0.1% aniline blue in phosphate buffer for visualizing callose. Pistil sections were observed under a fluorescence optical microscope as described above (Ex-Max 490 nm/Em-Max 525 nm).

### Statistical Analysis

Differences between sample sets (PAs content, TGase activity, and ELISA-based quantification of isopeptide (N*ε*-(*γ*-glutamyl)-lysine) content were determined by one-way or two-way ANOVA, with a threshold P-value of 0.05, using GraphPad Prism (v. 5.01 for Windows; GraphPad Software, San Diego, CA, United States). Where not differently reported, three technical replicates were performed.

## Results

### Temperature Affects Pollen Performance in Compatible and Incompatible Crosses of *C. clementina*

To verify the effect of optimal and non-optimal temperatures (25 and 15°C) on pollen tube growth under compatible/incompatible conditions in *C. clementina*, pollinated pistils were tested 10 days after pollination. Aniline blue staining was performed to visualize the growth of pollen tubes in the pistils. Dissection of pistils in three regions, *i.e.* stigmatic surface, style, and ovary region, allowed to compare pollen tube growth in compatible and incompatible crossings at 25°C (**Figures 1A–F**) and 15°C (**Figures 1G–N**). In incompatible (self-pollinated) crosses at 25°C the stigmatic region showed only few pollen tubes growing inside it (**Figure 1A**); some pollen tubes could be observed in underlying regions at level of style (**Figure 1B**) and were characterized by the accumulation of callose at the apex (arrow). No pollen tubes were observed at the ovary level (**Figure 1C**). On the contrary, the stigma of the same crossing (self-pollinated) at 15°C showed several pollen tubes growing inside it (**Figure 1G**). Thus, at 25°C, incompatible pollen tubes stopped their growth in the upper part of the style (**Figure 1B**, arrow), and no tube reached the ovary (**Figure 1C**). Therefore, the absence of pollen tubes in the median regions of the style suggested that the SI mechanism had blocked their growth. On the contrary, the same incompatible crossings at 15°C allowed the elongation of pollen tubes in the stigma (**Figure 1G**) and in the median part of styles (**Figure 1H**, arrows) as also shown by the detail of a cross section of the stylar canal (**Figure 1H**, arrows). The pollen tubes could then be detected up to the base of ovary (**Figure 1I**, arrow). When plants were kept at a temperature of 15°C, the incompatible (self-pollinated) samples were quite similar to compatible (cross-pollinated) and generally similar to compatible cross-pollinated samples observed at 25°C. Compatible cross-pollinated at 25°C (**Figures 1D–F**) and compatible cross-pollinated at 15°C (**Figures 1L–N**) showed numerous pollen tubes on the stigmatic surface, as well as many tubes elongating and reaching the ovary. Several pollen tubes were observed while penetrating the stigmatic surface (**Figures 1D, 1L**), while other tubes were detected in the median portion of styles (**Figures 1E, M**). Pollen tubes were clearly visible in sections taken at ovary level (**Figure 1F**, arrows and **Figure 1N**, arrow). The presence of pollen tubes along the entire stylar canal up to the ovary clearly showed that the SI response was absent. Pollen tubes reaching the ovary were counted and are reported in **Supplementary Material 2**.

### TGase Activity and Localization Differs in Self- and Cross-Pollinated Pistils at Different Temperatures

**Figure 2:**
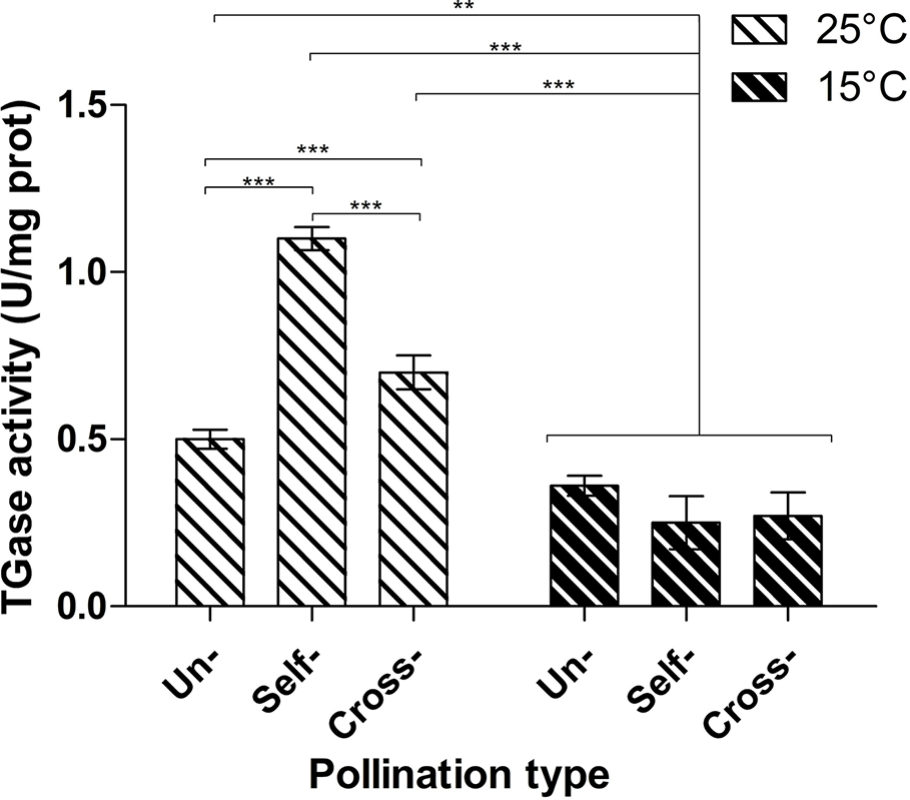
Microplate assay for TGase activity. TGase activity was tested in unpollinated, self-pollinated and cross-pollinated pistils at 25°C and at 15°C. TGase activity is expressed as units (U) of specific activity per mg of protein. Means ± S.D. of three experiments analyzed in triplicate are reported. The data were analyzed by one-way ANOVA and Tukey’s post-test. A p value < 0.05 was regarded as statistically significant. **p < 0.01. ***p < 0.001.

TGase activity was determined by the microplate assay in unpollinated, cross- and self-pollinated pistils at 25°C as well as in cross- and self-pollinated pistils at 15°C (**Figure 2**). The assay was performed by checking the incorporation of biotin-cadaverine in dimethylcasein and showed that TGase activity was higher at 25°C than 15°C (**Figure 2**). Moreover, at 25°C the activity in self-pollinated pistils increased sharply and was *ca*. 1.65-fold higher than in cross-pollinated ones at the same temperature and 2-fold higher than in unpollinated pistils. By comparing the self-pollinated pistils at 25°C and 15°C, enzymatic activity was about 4.5-fold higher at 25°C as compared to self-pollinated ones at 15°C. At 15°C there were no differences between unpollinated, self- and cross-pollinated pistils.

**Figure 3:**
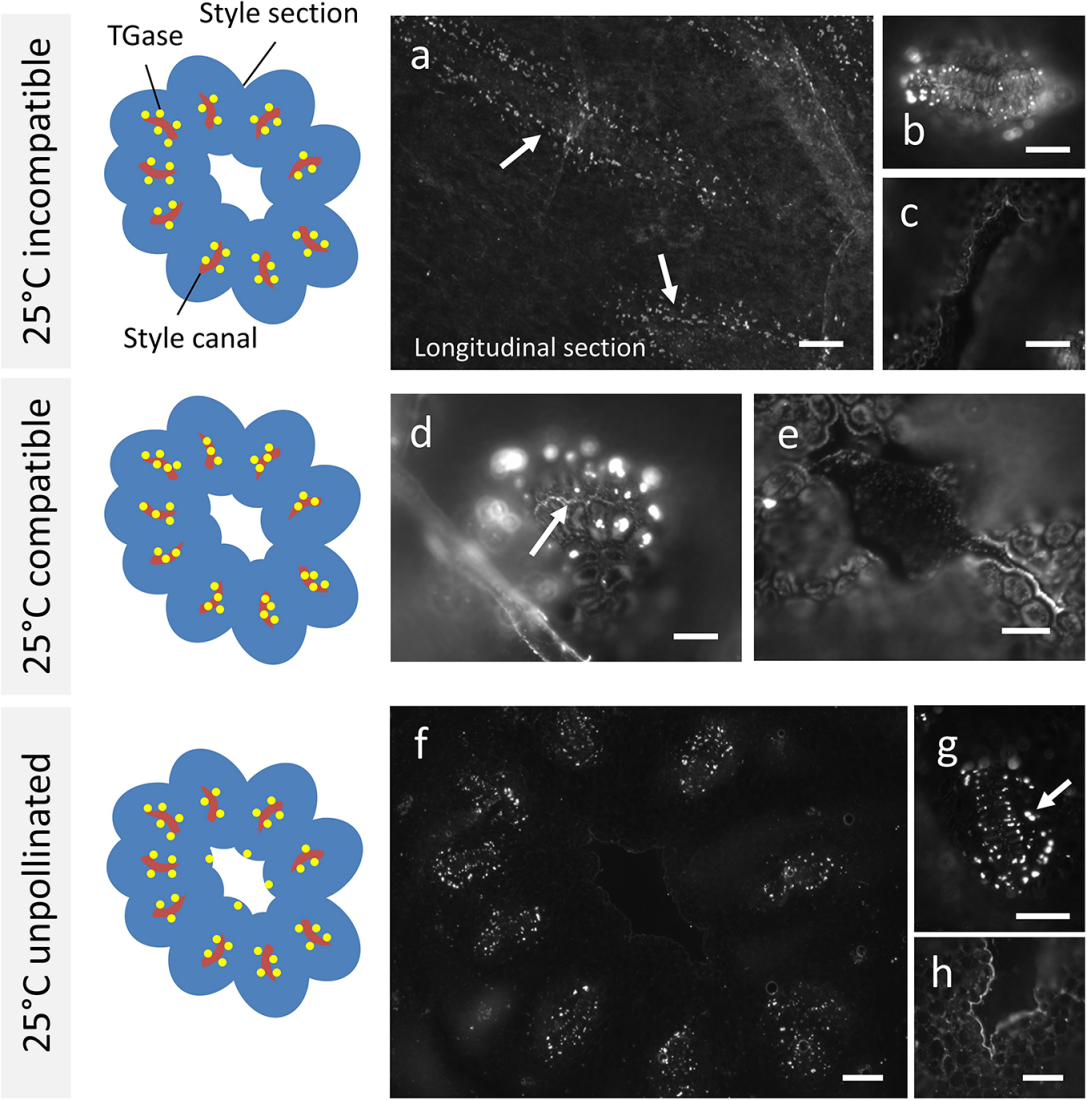
Immunolocalization of TGase during pollen germination in planta at 25°C. Labeling with anti-TGase by fluorescence microscopy in pollinated, unpollinated, compatible, and incompatible pistils at 25°C 10 days after pollination of flowers. For each presented case, the drawing on the left shows schematically the cross section of style (in blue) with the stylar canals (highlighted in red) and the distribution of TGase (yellow spots). **(A)** In the incompatible cross, TGase is found outside the style canal, as shown by the longitudinal section. **(B)** Detail of a cross-section of a style canal, with clear localization of TGase around the style canal. **(C)** The style canal does not show the TGase signal. **(D)** In the compatible cross, an accumulation of TGase can be seen inside the style canal (arrow) as well as outside it. **(E)** The central duct is not labeled. **(F)** When the pistil is unpollinated, the TGase signal is distributed outside the style canal, but not inside it. **(G)** Detail of a single style canal. **(H)** In a few cases, a weak TGase signal could be observed in the central duct. Scale bars: 100 μm.

The analysis of TGase distribution in *Citrus* samples crossed at 25°C revealed that the incompatible sample (**Figure 3A**) showed immunoreactive proteins at the edge of cells delimiting the stylar canals (arrows). This specific section is longitudinal. The signal does not appear to be distributed inside the style canal but rather on the outer edge of cells delimiting the canal, as shown by the detail in **Figure 3B** (cross-section). The central duct did not give any signal (**Figure 3C**), nor did the cells outside the style canal. In the case of compatible crosses, the picture was quite different. TGase signal was immunodetected prominently inside the style canal (**Figure 3D**, arrow), as well as around the style canals in the form of well-defined spots. Again, the central duct did not show any signal (**Figure 3E**). The results of the incompatible crosses were very similar to those of unpollinated styles (**Figure 3F**). Again, labeling with anti-TGase targeted the external side of cells delimiting the style canals (see detail in **Figure 3G**, arrow). Sometimes the central duct showed a slight labeling (**Figure 3H**), but this was not a very frequent case.

**Figure 4:**
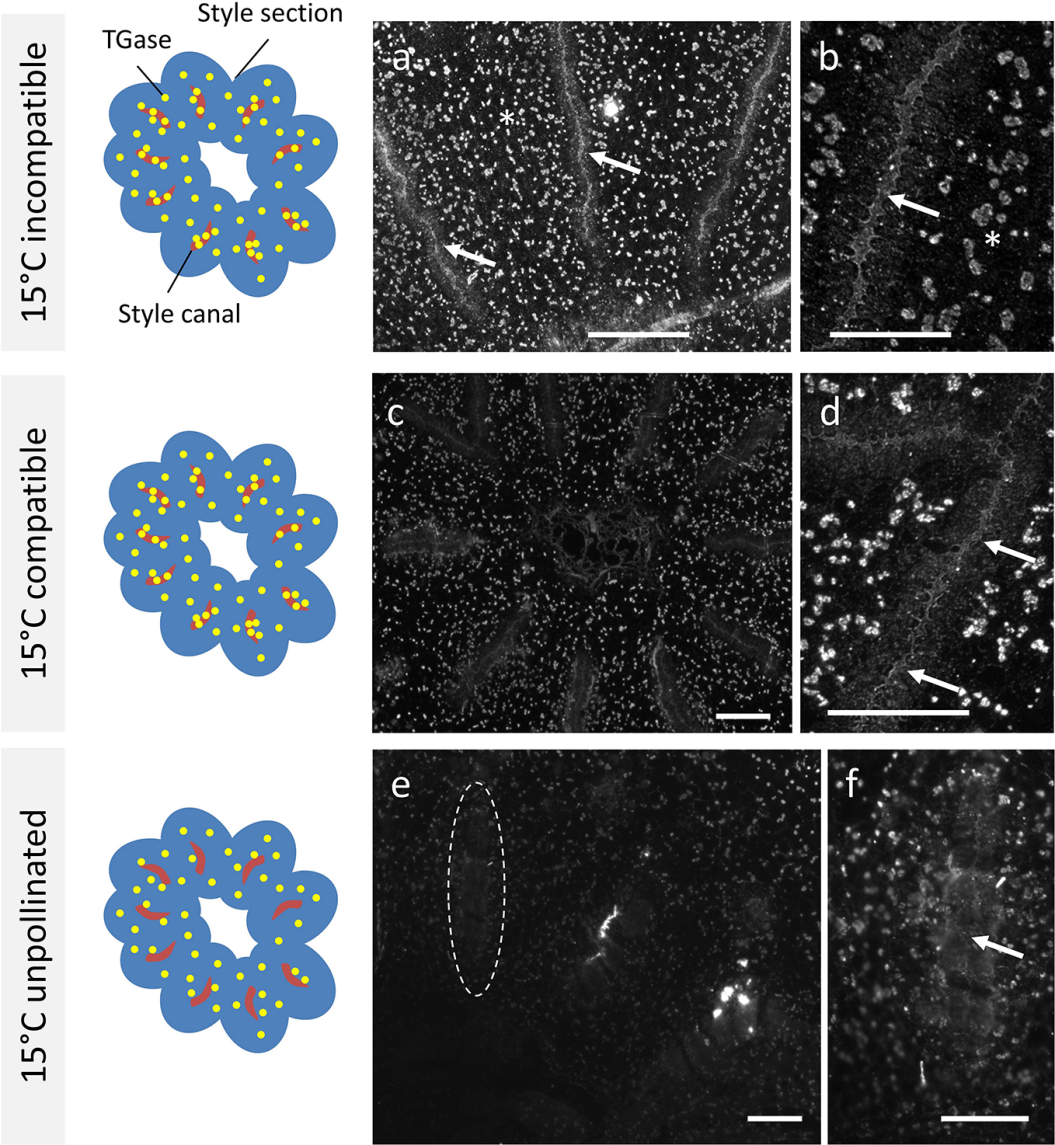
Immunolocalization of TGase during pollen germination in planta at 15°C. Distribution of TGase in compatible, incompatible, pollinated, and unpollinated styles, crossed at 15°C. Again, in each reported case the left-hand drawing schematically depicts the style cross-section (in blue), the stylar canals (in red) and the pattern of TGase (yellow spots). **(A)** In the incompatible crosses, an intense TGase signal can be observed in the style canal (arrows) but also externally in the form of diffuse spots (asterisks). **(B)** Detail of a style canal with clear accumulation of TGase internally (arrow). **(C)** In the compatible crosses, a clear TGase signal was observed inside the style canal and TGase was also distributed externally as diffuse spots. **(D)** Detail of a style canal with an intense internal TGase signal (arrows) and with a diffuse external signal. **(E)** In unpollinated styles **(E)** no TGase signal was present inside the style canal (indicated by the dotted oval); a diffuse faint signal was observed externally. **(F)** Detail of a style canal with no signal inside. Scale bars: 100 μm.

When *Citrus* samples were analyzed after crossing at 15°C, the results were not in line with results at 25°C. In the case of incompatible crosses (**Figure 4A**), the signal produced by the anti-TGase antibody could be clearly observed within the stylar canals (arrows). The signal filled precisely the entire inner surface of the stylar canal. As a further difference, a widespread signal was also present in cells outside the stylar canals in the form of well-defined spots (asterisk). The detail in **Figure 4B** unequivocally shows the TGase signal within the stylar canal (arrow). In the case of compatible crosses (**Figure 4C**), the picture that emerged was almost comparable to the incompatible case. Indeed, the stylar canals showed a clear internal signal and the cells surrounding the stylar canal were also extensively labeled. The detail in **Figure 4D** highlights both the signal inside the stylar canal (arrows) and the very diffuse signal in the surrounding cells. The situation observed in the case of unpollinated pistils was very specific and distinct from other cases. First, the stylar canals were not labeled internally (**Figure 4E**, see stylar canal highlighted by the dotted circle). The detail in **Figure 4F** still highlights the absence of a signal inside the stylar canal (arrow), but also shows the widespread labeling of surrounding cells.

### TGase Increases Protein Cross-Linking and Bound PA Content, Mostly in Incompatible Pollinated Pistils of *C. clementina*

**Figure 5:**
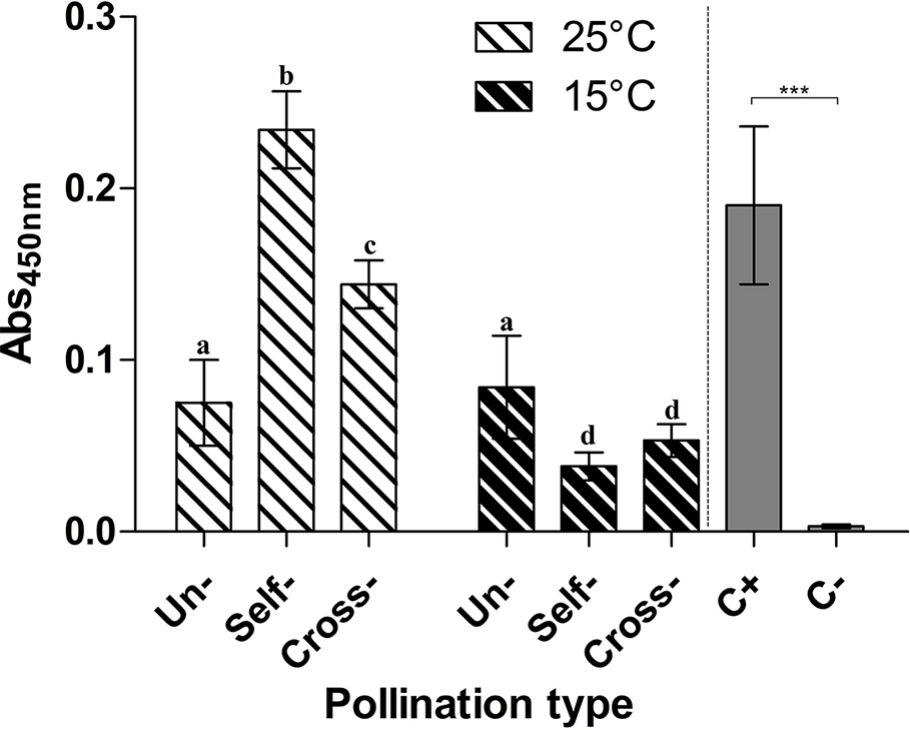
Immunoquantification of pollen TGase cross-linked products. ELISA assay for the TGase isopeptide product N^*ε*^-(**γ**-glutamyl)-lysine. The presence of N^*ε*^-(**γ**-glutamyl)-lysine was tested in unpollinated, self-pollinated, and cross-pollinated pistils at 25°C and at 15°C, by the 81D4 monoclonal antibody. As a control, casein treated (C+) or not (C−) with TGase was used in the same ELISA assay. Means ± S.D. of three experiments analysed in triplicate are reported. Samples indicated with the same letters are not significantly different. The data were analyzed by two-way ANOVA and Tukey’s post-test. A p value < 0.05 was regarded as statistically significant. Casein treated with TGase (C+) was significantly different from casein not treated with TGase (C−) as demonstrated by one-way ANOVA. ***p < 0.001.

The presence of the TGase reaction product, the isopeptide N^*ε*^-(*γ*-glutamyl)-lysine, was investigated in unpollinated, cross- and self-pollinated pistils at 25°C as well as in cross- and self-pollinated pistils at 15°C. Results showed that at 25°C, the TGase reaction product was higher when compared to 15°C (**Figure 5**). Moreover, at 25°C in self-pollinated pistils the isopeptide was *ca*. 2-fold higher than in cross-pollinated pistils at the same temperature and 2.6-fold as compared to unpollinated pistils. In self-pollinated pistils, the isopeptide content was about 6-fold higher at 25°C than at 15°C, indicating that the SI response occurring at 25°C enhanced the formation of TGase products. At 15°C, there were no differences between unpollinated, self- and cross-pollinated pistils.

**Figure 6:**
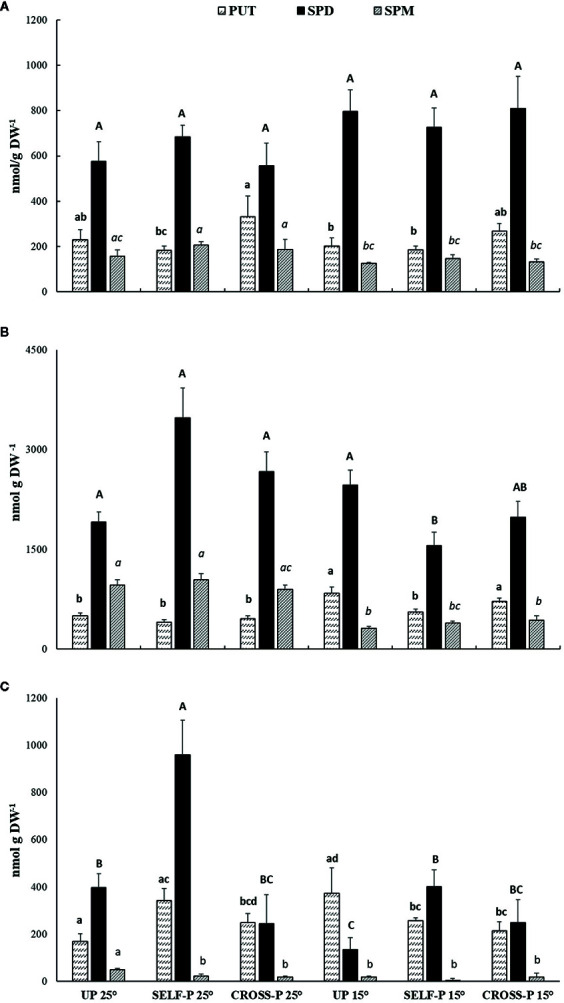
HPLC analysis of PAs content. Free **(A)**, soluble-conjugated **(B)** and insoluble-bound **(C)** PAs in unpollinated, self-, and cross-pollinated pistils of *C. clementina*. The data were analyzed by two-way ANOVA test, with Bonferroni post-test. Values of p < 0.05 were considered statistically significant. Different letters indicate significant differences at p < 0.05. For each PA, a different font was used (Put, lowercase; Spd, uppercase; Spm, italics). Means ± S.D. of three experiments analyzed in triplicate are reported.

Like the cross-linked protein products, PAs, well-known substrates of TGase, were also determined in pistils of plants grown at 25°C and 15°C (**Figures 6A–C**).

Spd was the most abundant PA in the free form (**Figure 6A**) and did not show significant differences among samples. Few significant differences among samples were also observed for Put and Spm.

Soluble-conjugated PAs were the most abundant (**Figure 6B**); in this form, Spd reached levels ranging from 1.5 to 3.4 µmol/g DW, with the highest concentrations in self-pollinated styles grown at 25°C. In these samples, Spd levels were significantly higher than in self-pollinated pistils grown at 15°C. Spm concentrations in the soluble-conjugated form were in general lower in pistils of plants grown at 15°C compared to their counterparts grown at 25°C.

Self-pollinated pistils showed the highest concentration of Spd in the insoluble-bound form (**Figure 6C**), with levels that were more than double those of both unpollinated and cross-pollinated ones grown at the same temperature. A pronounced decrease in bound Spd levels was observed in unpollinated and self-pollinated pistils grown at 15°C compared to their counterparts grown at 25°C, while no significant differences between the two temperature regimes were observed in the case of cross-pollinated pistils (**Figure 6C**).

## Discussion

Previous reports indicated that in *C. clementina* temperature has a strong effect on flower and ovary development, pollen germination, and pollen tube growth (Distefano et al., 2018). In the present investigation, we detected a correlation between type of pollination (compatible *vs* incompatible), temperature, TGase activity and distribution, and PA pattern. Exposure to different temperatures during flower bud development altered, in fact, the magnitude of the SI reaction, thereby affecting the elongation of pollen tubes, as well as the number of pollen tubes reaching the ovary. In particular, we observed that at 15°C the self-pollinated samples behaved like the self-compatible ones, while remaining incompatible at 25°C. Histological analyses indicated that pollen rejection occurred according to the GSI mechanism, as pollen tubes developed, but their growth was blocked in the upper or middle part of the style. Pollen tubes were unable to reach the base of style, whereas in compatible pollination a significant number of pollen tubes reached the ovary. SI also caused alterations in pollen tube morphology, which look twisted and showed accumulation of callose in the apical region, as highlighted by aniline blue staining. The formation of callose plugs at the tip of SI pollen tube is a feature shared by other SI systems in which these callose formations were related to the interruption of the pollen tube growth in incompatible fertilization. For example in *Pyrus communis* pollen tube penetration into the gynoecium reached only the upper first third of the style length, whereas in the compatible system the tube grew up to the ovule without any sign of apical callose plugs (Del Duca et al., 2010). Similar callose plugs are present in SI pollen tubes in *Citrus grandis* (Gentile et al., 2012), *Zea mays* (Lu et al., 2014), *Petunia hybrida* (Oloumi and Rezanejhad, 2009), and in *Nicotiana alata* (Lush and Clarke, 1997).

The arrest of pollen tube growth could be linked to increased TGase activity, which promoted the formation of cross-link aggregates, as clearly shown by results obtained with the anti-isopeptide antibody 81D4. These aggregates were lower in compatible pollinated- and in unpollinated pistils, suggesting the involvement of TGase in processes like cell wall stiffening and cytoskeleton modifications that contribute to block pollen tube growth (Del Duca et al., 2010; Mandrone et al., 2019). At 15°C, the self-pollinated pistils did not show any enhancement of TGase product formation, suggesting that genotype is not sufficient to determine a TGase-based response, while a genotype × temperature interaction is necessary for the involvement of this enzyme. Since TGase is a Ca^2+^-dependent enzyme, its activity might be controlled downstream by the modulation of free Ca^2+^, which increases up to mM concentrations under stress conditions, including the SI response (Griffin et al., 2002; Ranty et al., 2016). As TGase activity takes place from 20 nM Ca^2+^ upwards, massive protein cross-linking, and consequent formation of protein aggregates, as quantified in the present work, can easily occur in cross-pollinated samples.

Increasing free cytosolic Ca^2+^ concentration also alters the cytoskeleton structure during SI, as reported in *Papaver rhoeas* and *Pyrus bretschneideri*. Free cytosolic Ca^2+^ increase triggers F-actin depolymerization, ROS increases, and the stimulation of caspase-like activities, leading pollen to programmed cell death (PCD) (Wilkins et al., 2014; Chai et al., 2017). Moreover, in *Pyrus* self-incompatible pollen, S-RNase interacts directly with F-actin, enhancing the PCD signaling (Wilkins et al., 2014; Chai et al., 2017). In the same plant model, S-RNase also alters lipid signaling, *i.e.* phosphatidic acid, which initially counteracts the effects of S-RNase (Chen et al., 2018). Recently, PAs were also demonstrated to trigger a rapid increase in phosphatidic acid in *Arabidopsis thaliana* due to a transient efﬂux of K^+^ and the activation of a specific phospholipase D variant (Zarza et al., 2019), that dramatically elevates phosphatidic acid levels within minutes of SI elicitation. However, the precise mechanism of action of phosphatidic acid during SI remains an issue yet to be clarified (Chen et al., 2018).

A strong increase in intracellular Ca^2+^ during SI has been demonstrated for several plant models, and recently also in *Citrus*, where a significant increase of free cytosolic Ca^2+^ was highlighted within 15 min after induction of the SI response with stylar crude protein extracts. Moreover, by laser capture microdissection coupled to microarray, Caruso and co-workers (Caruso et al., 2012) identified candidate genes involved in self-pollen rejection which have not been previously associated with SI. In detail, three uncharacterized genes encoding for aspartic acid-rich proteins (CcASP-RICH) are strongly activated concomitantly with the arrest of pollen tube growth following the SI response. CcASP-RICH are small proteins containing 79, 53, or 74 amino acids. Although they do not show sequence homology with other well known Ca^2+^ interacting proteins (*i.e.* calsequestrin, calreticulin, and calmodulin), the Asp-rich proteins share with those proteins the high content in aspartate residue which has been related with the protein ability of Ca^2+^-binding (Shin et al., 2000). Due to their primary structure and the abundance in aspartic acid residues, these proteins might function as Ca^2+^ binding proteins (Distefano et al., 2009; Caruso et al., 2015). As Ca^2+^ levels and distribution inside the pollen tube play a decisive role during pollen–pistil interaction (Ge et al., 2007), it is reasonable to hypothesize that the observed increased expression of CcASP-RICH in the SI response could lead to an impairment in the levels of free cytosolic Ca^2+^. They might thus contribute to switch off the signal cascade usually induced by the increase in cytosolic Ca^2+^ concentration or to the alteration of Ca^2+^ gradient needed for pollen tube elongation, as reported for other examples of GSI (Wilkins et al., 2014; Chai et al., 2017; Chen et al., 2018). However, further work at the protein level will be necessary for understanding the role of the candidate genes during self-pollen recognition. Moreover, microarray experiments performed by Caruso and co-workers (Caruso et al., 2012) allowed to identify the unigenes represented in the Affymetrix Citrus GeneChip that do not cover the whole transcriptome, and consequently other strategies such as RNA-seq might help to improve the transcriptome coverage to identify additional genes implicated in self-pollen recognition.

**Figure 7:**
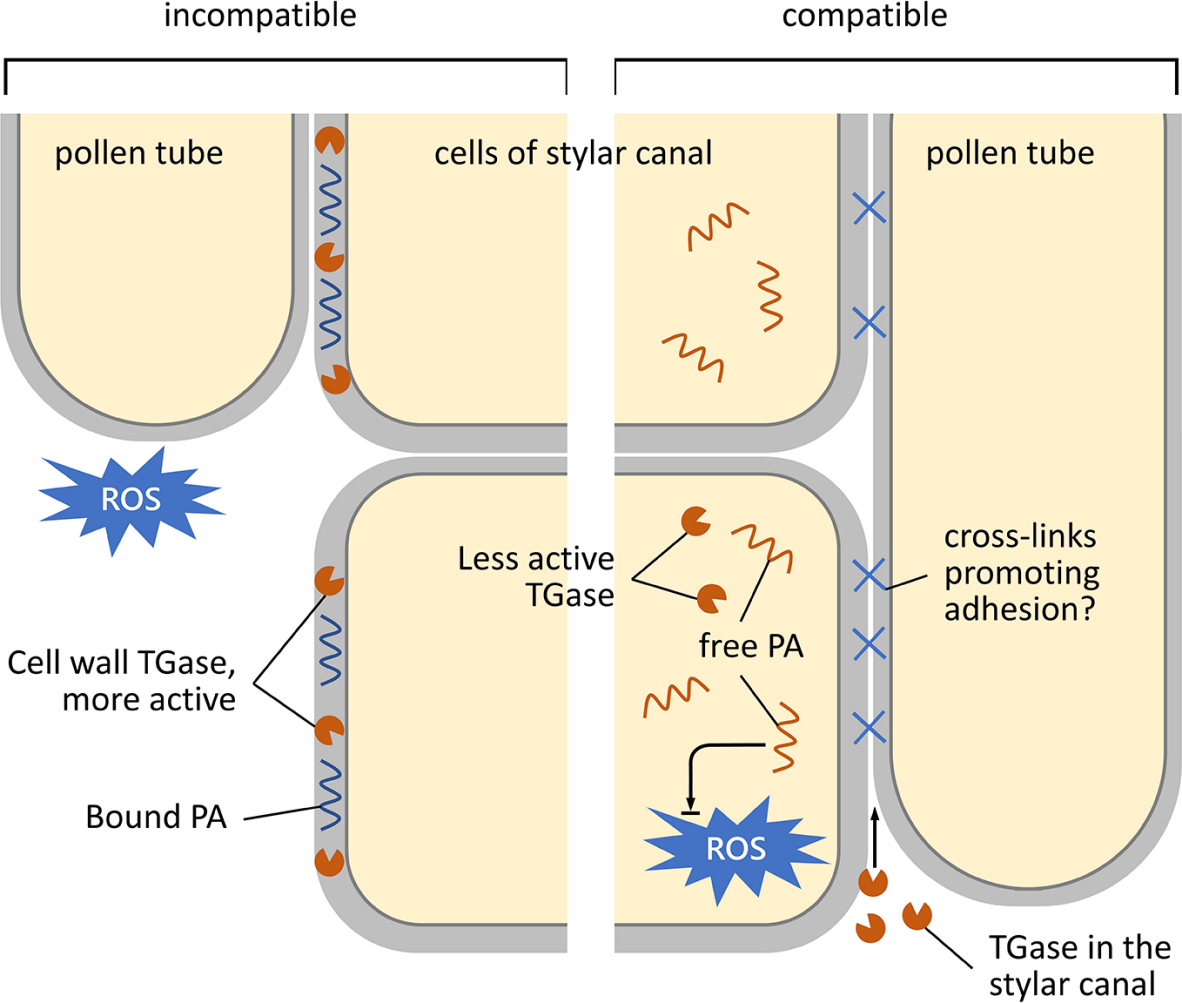
Schematic representation of the growth and adhesion of pollen tubes during their pathway through the stylar canal. Left, incompatible condition; right, compatible condition. In the case of the incompatible reaction, TGase accumulates in the cell walls of cells of the stylar canal. Here, TGase is enzymatically active and results in a higher percentage of bound PAs. It is assumed that bound PAs serve to strengthen the cell wall by preventing damage to the stylar cells during the rejection of pollen tubes (caused, for example, by an increase in ROS). In the compatible situation (right), TGase is located in the cytoplasm of stylar cells in a less or non-active form. Therefore, PAs are more in the free form and could serve to counteract any increase in ROS. The TGase present in the stylar canal would in this case play a role in promoting adhesion between stylar cells and pollen tubes in order to promote fertilization.

Besides TGase activity regulation by tuning free cytosolic Ca^2+^, enzyme localization represents another regulatory strategy. In this work, we show that in compatible crosses at 25°C, TGase localized inside the stylar canal, whereas in the incompatible ones it was present on the outer surface. The localization of TGase inside the stylar canal could promote pollen tube adhesion to the stylar cells, favoring the cross-talk between male and female counterparts and the growth of pollen tubes. This hypothesis is coherent with previous results in apple, which highlighted the role of secreted TGase in the adhesion of the pollen tube to stylar cells, allowing style anchorage and subsequent tube migration (Di Sandro et al., 2010). These features are shared with mammalian tTGases, which play a pivotal role for cell adhesion to the extracellular matrix and have been, therefore, defined as “natural biological glue” (Griffin et al., 2002). Consequently, we can suggest the following model of how TGase can act in cases of incompatibility and compatibility in *C. clementina* (**Figure 7**). In the incompatible crossbreeding (**Figure 7**, left panel), TGase is likely to be present in the cell wall of the stylar canal cells in active form; in this way, TGase could strengthen the cell wall by making it more resistant against mechanisms (such as ROS production) that block pollen tube growth. On the other hand, in the compatible case (**Figure 7**, right-hand panel), TGase would be less active and localized in the cell cytoplasm; here, free PAs contribute to homeostasis of ROS. The TGase secreted in the stylar canal could hypothetically contribute to the formation of cross-links between the stylar canal cells and the pollen tube, thus promoting adhesion and growth.

PAs can affect TGase activity in two ways: (i) they are physiological substrates of TGase, and (ii) they are regulators of cytosolic Ca^2+^ levels (Alcázar et al., 2010), leading to the above-mentioned rearrangements of the actin cytoskeleton mediated by TGase (Aloisi et al., 2017).

Concerning the pattern of PAs, results indicated that, among the free PAs, only Put was related to the type of pollination, significantly decreasing during the SI response at 25°C, whereas free Spd and Spm did not show remarkable changes. Similar changes in Put, but not in Spd and Spm, have been related to several stress conditions (Ruiz-Carrasco et al., 2011). Free PAs behave as ROS scavengers, as demonstrated also in *C. reticulata* where Spm pre-treatment caused accumulation of endogenous PAs and, accordingly, a more effective ROS scavenging (Velikova et al., 2000; Shi et al., 2010). Given the role of PAs, the lower level of Put during SI could be related to cell damage, being the reduced Put level not able to counteract the ROS action. Even if ROS are necessary for pollen tube growth, an impaired ROS homeostasis, leading to their exceeding accumulation, causes damage to cell structures (Daher and Geitmann, 2011; Speranza et al., 2012; Sewelam et al., 2016), hence contributing to block pollen tube growth. Moreover, the lower level of free Put in self-pollinated style could be the result of PAs’ metabolism (synthesis of Spd, degradation or linkage to other molecules) as also suggested by the concomitant significant increase in soluble-conjugated fraction.

In *C. clementina,* soluble-conjugated PAs were the most abundant, and changes in Spd levels were related to the temperature-dependent SI response. These compounds originate from the linkage of PAs either to phenylpropanoids or to peptides and/or proteins with molecular weight lower than 5 kDa. The phenylpropanoid–PAs conjugates, also called hydroxycinnamic acid amides or phenolamides, are specialized metabolites and mainly accumulate in reproductive organs. A recent paper reported that high concentrations of hydroxycinnamoyl–spermidine conjugates were found in nectar and pollen of several Angiosperms (Palmer-Young et al., 2019). Phenolamides are involved in pollen cell wall organization (Ito et al., 2007; Yang et al., 2007; Grienenberger et al., 2009) and fertilization (Lam et al., 1992; Elejalde-Palmett et al., 2015) and serve as defense compounds in biotic interactions.

It is well known that most of the cellular Spd pool is localized in the cell wall compartment (Bokern et al., 1995; Biasi et al., 2001). In the light of PA interactions with cell wall molecules, it is possible that soluble-conjugated-Spd influences pollen germination *via* a structural effect on the cell wall, thus enhancing its strength. This, in turn, could be especially important in the SI response when pollen tube apical plugs are formed.

In *C. clementina*, self-pollination at 25°C also caused a remarkable increase in insoluble-bound Spd, with levels higher than those of both cross-pollinated pistils at the same temperature and self-crossed pistils at 15°C. These PAs derive from the binding of free PAs or soluble-conjugated PAs to high molecular mass partners (such as proteins) or cell wall molecules like hemicelluloses and/or lignin (Antognoni and Bagni, 2008). These results agree with the increase in TGase activity occurring during the SI reaction, also reported in *C. grandis* (Gentile et al., 2012) and *Pyrus communis* (Del Duca et al., 2010; Mandrone et al., 2019). In these experimental models, incompatible pollination resulted in enhanced TGase activity and levels of conjugated-PAs when compared to cross-pollination.

The presence of PAs and TGase in the cell wall is not surprising. Indeed, literature data suggest that TGase could be involved in the organization of cell walls. As a support to these hypotheses, it should be mentioned that in petals of *Nicotiana tabacum* (Della Mea et al., 2007) and in the pollen tube of *Malus domestica*, TGase and its products were actually localized in the cell wall (Di Sandro et al., 2010).

In conclusion, we show here that in *C. clementina* the involvement of TGase during the SI response is similar to the one described for members of the Rosaceae family (Del Duca et al., 2010) and in other Rutaceae species (Gentile et al., 2012). This suggests that the molecular mechanism underlying SI could be a common feature shared by unrelated species belonging to different families. What remains to be established is the contribution of each partner (pollen and/or pistil) to the complex scenario of the PA–TGase interplay and how temperature influences SI. Understanding these aspects could have a practical and economic impact, insofar as sprayed PAs are able to increase fruit set percentage and reduce SI (Androulakis and Loupassaki, 1990; Sayyad-Amin et al., 2018).

## Data Availability Statement

The raw data supporting the conclusions of this article will be made available by the authors, without undue reservation.

## Author Contributions

SD, GC, IA, GD, and AG designed the experiment. SB and GD performed the temperature treatment for flower samples. IA, FA, GP, LP, and CF performed the experiments, analyzed the data and prepared the figures. SD, GC, and IA planned the research and interpreted the data. SD and IA wrote the article with contributions from all other authors.

## Funding

This work was supported by PRIN 2015 ISIDE (Investigating Self Incompatibility Determinants in fruit trees) (http://prin.miur.it/) to SD, AG and GC.

## Conflict of Interest

The authors declare that the research was conducted in the absence of any commercial or financial relationships that could be construed as a potential conflict of interest.
