# Supplementary material for: Temperature-Dependent Compatible and Incompatible Pollen-Style Interactions in Citrus clementina Hort. ex Tan. Show Different Transglutaminase Features and Polyamine Pattern
Source: Front Plant Sci. 2020 Jul 8;11:1018. doi: 10.3389/fpls.2020.01018 (PMC7360793; doi:10.3389/fpls.2020.01018)
Supplement: Supplementary file 1 [file Presentation_1.pptx]

## Slide 1
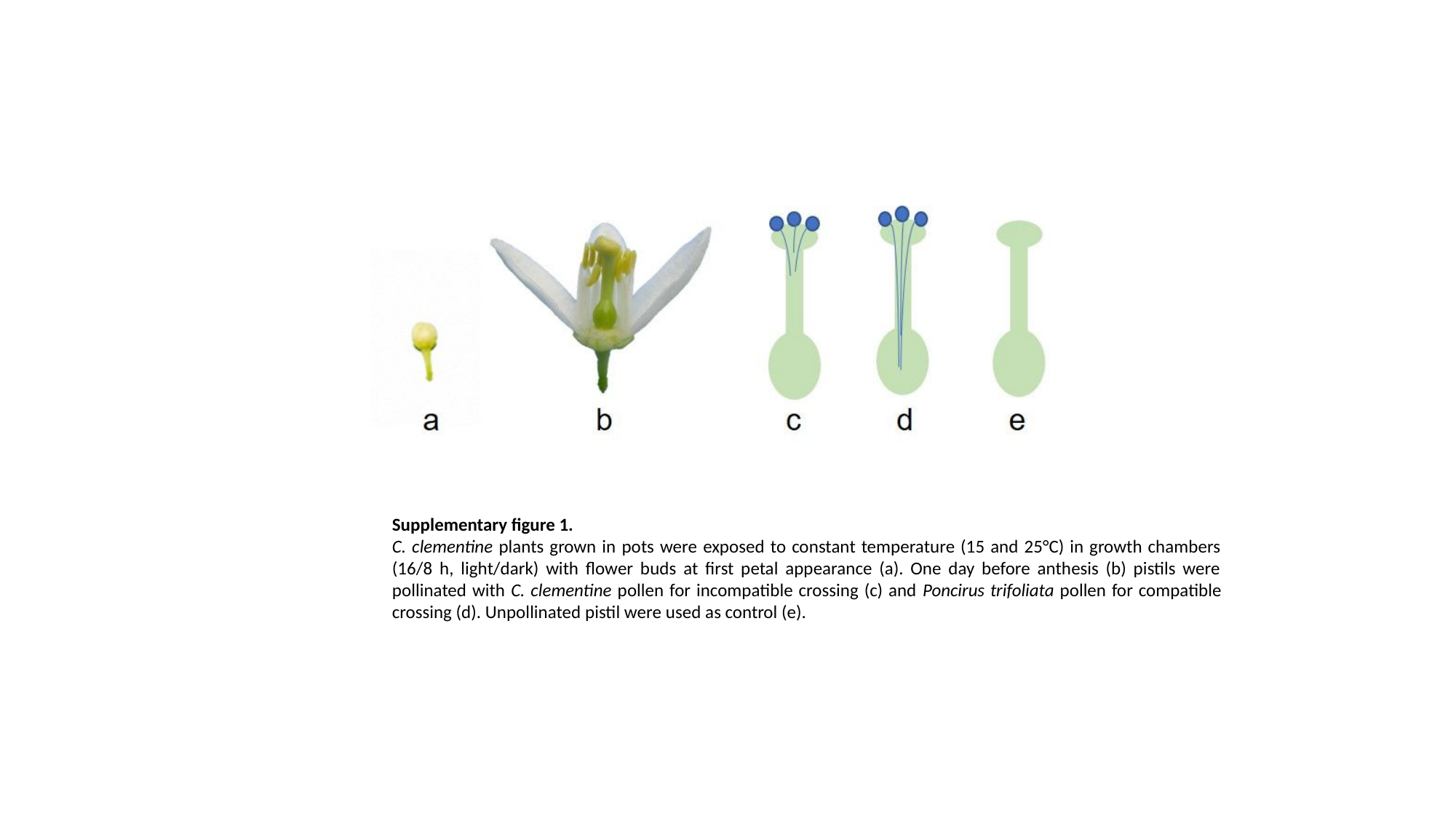

Supplementary figure 1.
C. clementine plants grown in pots were exposed to constant temperature (15 and 25°C) in growth chambers (16/8 h, light/dark) with flower buds at first petal appearance (a). One day before anthesis (b) pistils were pollinated with C. clementine pollen for incompatible crossing (c) and Poncirus trifoliata pollen for compatible crossing (d). Unpollinated pistil were used as control (e).
