# Supplementary material for: Temperature-Dependent Compatible and Incompatible Pollen-Style Interactions in Citrus clementina Hort. ex Tan. Show Different Transglutaminase Features and Polyamine Pattern
Source: Front Plant Sci. 2020 Jul 8;11:1018. doi: 10.3389/fpls.2020.01018 (PMC7360793; doi:10.3389/fpls.2020.01018)
Supplement: Supplementary file 2 [file Presentation_2.pptx]

## Slide 1
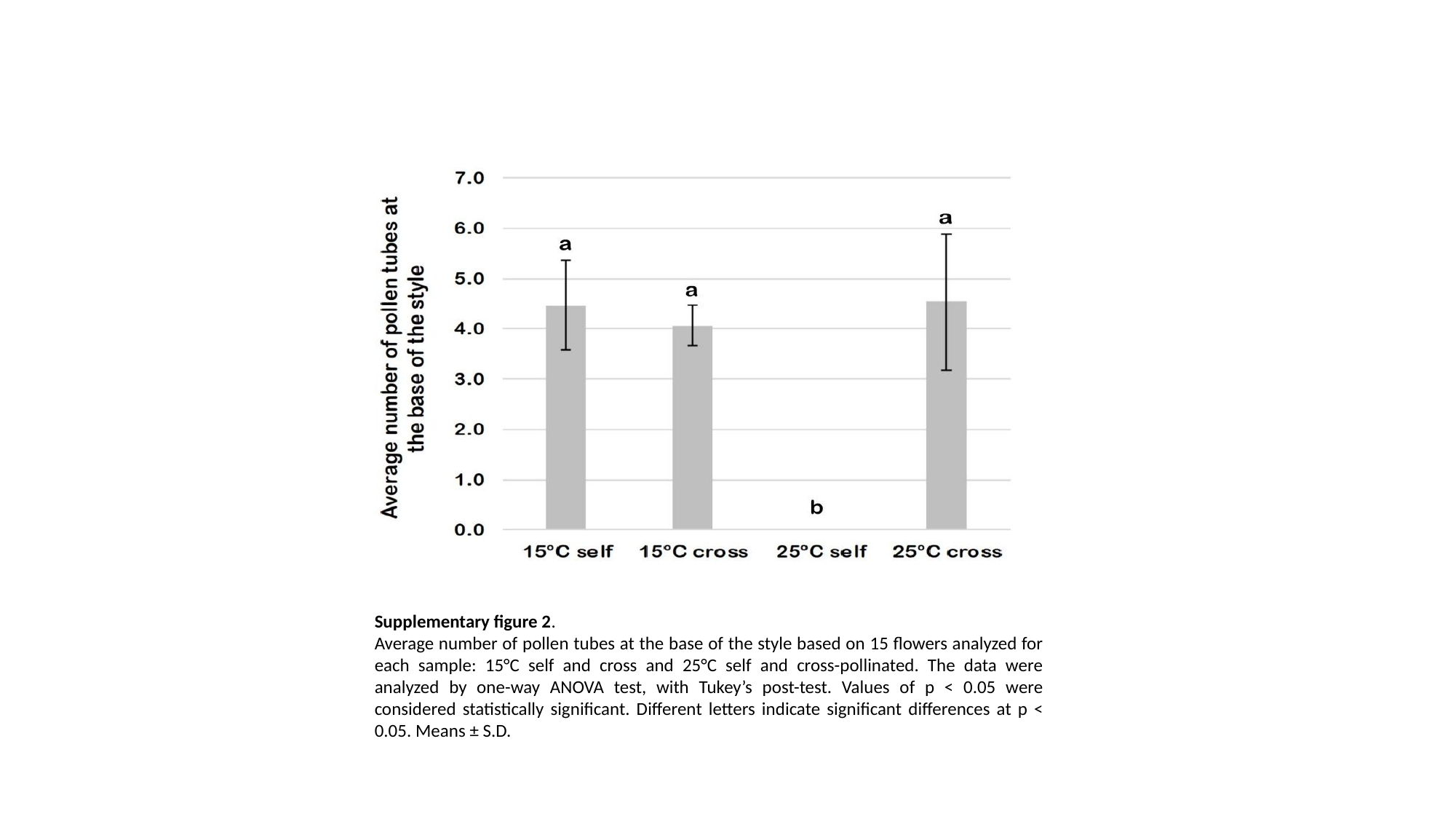

Supplementary figure 2.
Average number of pollen tubes at the base of the style based on 15 flowers analyzed for each sample: 15°C self and cross and 25°C self and cross-pollinated. The data were analyzed by one-way ANOVA test, with Tukey’s post-test. Values of p < 0.05 were considered statistically significant. Different letters indicate significant differences at p < 0.05. Means ± S.D.
